# Changes in Physical Function and Locomotive Syndrome Status in the Postoperative Period Following Total Hip and Knee Arthroplasty

**DOI:** 10.7759/cureus.93618

**Published:** 2025-09-30

**Authors:** Hiroto Takenaka, Kunio Ida, Miho Kawamura

**Affiliations:** 1 Department of Physical Therapy, Faculty of Health and Medical Sciences, Tokoha University, Hamamatsu, JPN; 2 Department of Rehabilitation, Kasugai Orthopedic Asahi Hospital, Kasugai, JPN

**Keywords:** locomotive syndrome (ls), long-term outcomes, physical function, total hip arthroplasty (tha), total knee arthroplasty (tka)

## Abstract

Objective: This study aims to examine changes in physical function and locomotive syndrome (LS) status at different postoperative stages after total hip arthroplasty (THA) and total knee arthroplasty (TKA), emphasizing long-term functional maintenance and prevention of mobility decline.

Methods: This cross-sectional study included 118 THA or TKA patients categorized into four postoperative duration groups: short-term (<24 months, n=21), mid-term (24 to 59 months, n=29), long-term (60 to 119 months, n=32), and very long-term (≥120 months, n=36). Physical function was measured using handgrip strength, the five-chair stand (5-CS) test, one-leg standing time, usual walking speed, and a short physical performance battery. The LS status was assessed using the stand-up test, two-step test, and 25-question Geriatric Locomotive Function Scale (GLFS-25).

Results: Despite similar evaluation ages (73 to 74 years), the very long-term group had significantly longer 5-CS times (10.8±3.5 vs 8.5±2.4 seconds in short-term, p=0.04) and higher GLFS-25 scores (15.0±9.0 vs 8.5±5.7, p=0.005). Severe LS (stage 3) increased from 4.8% in the short-term to 30.6% in the very long-term group (p=0.04). In the THA subgroup analysis (n=94), these differences remained significant after adjusting for age at surgery, BMI, and bilateral surgery status (adjusted p=0.028 for 5-CS, p<0.001 for GLFS-25). Basic mobility functions such as walking speed and balance remained stable across periods. Bilateral surgery cases increased with longer postoperative duration, reaching 61.1% in the very long-term group (p=0.02).

Conclusions: Joint arthroplasty provides sustained benefits in basic mobility functions over extended periods. However, muscle power-dependent functions and LS severity show time-dependent deterioration, particularly beyond 10 years post-surgery. This decline persists after adjustment for confounders and occurs even in unilateral cases, indicating a true time-related phenomenon. The high prevalence of bilateral surgery in long-term follow-up likely reflects the natural history of hip dysplasia but does not fully explain functional decline. These findings underscore the importance of long-term monitoring and targeted interventions to maintain muscle strength, especially beyond 10 years post-surgery.

## Introduction

Total hip arthroplasty (THA) and total knee arthroplasty (TKA) are well-established surgical interventions for severe osteoarthritis that provide significant pain relief and functional improvement [1,2]. The long-term maintenance of physical function has become increasingly significant due to the rising life expectancy and growing number of patients undergoing joint arthroplasty at younger ages [3,4]. Understanding the trajectory of functional outcomes over extended periods is essential for the development of effective long-term rehabilitation strategies.

Although TKA and THA have been shown to improve physical function, postoperative fall rates remain high and are comparable to those observed in patients with severe knee and hip osteoarthritis [5]. Several studies have reported functional deficits in long-term follow-up after arthroplasty [6,7], with increased fall risk, particularly in later postoperative years [8]. These findings highlight the importance of continued monitoring and preventive intervention. Additionally, many patients who undergo TKA or THA are elderly, and age-related changes in the neural and muscular systems, along with age-related degenerative joint changes, can contribute to decreased mobility and increased long-term fall risk. These findings highlight the need for continuous monitoring of physical function during the postoperative period.

Preventing decline in mobility is crucial, and the concept of locomotive syndrome (LS) has been proposed as a measure [9]. This concept represents the state of requiring care due to mobility disorders or being at a high risk of needing care in Japan [10,11]. While previous studies have examined LS in arthroplasty patients up to two years post-surgery [12,13], there is limited understanding of how LS severity changes across different postoperative periods, particularly in the long term.

This gap in knowledge is especially significant given the aging population and the heightened expectation that functional performance can be preserved long-term to reduce fall-related fractures [14]. Understanding the temporal relationship between the postoperative duration and functional outcomes, including LS status, could provide crucial insights for developing targeted long-term rehabilitation strategies. Moreover, identifying specific periods of increased risk for functional decline could help optimize the timing and nature of preventive interventions. Therefore, this study aimed to investigate the changes in physical function and LS status across different postoperative periods after THA and TKA.

## Materials and methods

This cross-sectional study includes patients who underwent TKA or THA performed by orthopedic surgeons at a single institution. Eligible patients were those who were at least six months postoperative and were independent in daily activities during their visits from March 2022 to February 2023. Patients were excluded if they had difficulty walking, required a walker for daily activities, or showed cognitive decline (defined as a Mini-Mental State Examination [15] score of < 23 or the Hasegawa Dementia Scale-Revised [16] score of < 20). These exclusion criteria were established to ensure accurate performance-based assessments, as the functional tests require independent mobility and the ability to follow complex instructions. In total, 118 patients agreed to participate in the study and were evaluated. The baseline patient characteristics are shown in Table 1. Physical functions were assessed by 10 physical therapists who were randomly assigned to conduct the evaluations. These therapists discussed and standardized the assessment methods before beginning the evaluations. The assessment tools used in this study, including the 25-question Geriatric Locomotive Function Scale (GLFS-25) [17], Short Physical Performance Battery (SPPB) [18], and five-repetition chair stand (5-CS) test [19], are all publicly available and were used in accordance with their established guidelines for research purposes.

**Table 1:** Demographic and clinical characteristics of participants by postoperative duration group Values are presented as mean (SD) unless otherwise indicated. The p-values were calculated using the Kruskal-Wallis test (H statistic) for continuous variables and the chi-square test (χ² statistic) for categorical variables. *Significant difference between groups (p<0.05); THA: Total Hip Arthroplasty; TKA: Total Knee Arthroplasty

Factor	Group	Short (<24 months)	Mid (24 to 59 months)	Long (60 to 119 months)	Very long (120 months)	Test statistic (χ² or H)	p-value
Number		21	29	32	36		
Surgery (%)	THA	16 (76.2)	23 (79.3)	21 (65.6)	34 (94.4)	χ² = 8.90	0.03*
	TKA	5 (23.8)	6 (20.7)	11 (34.4)	2 (5.6)		
Age (in years)		74.0 (4.3)	73.86 (5.70)	73.3 (6.2)	73.7 (6.2)	H = 0.49	0.97
Age at initial surgery (in years)		72.7 (4.3)	70.4 (5.7)	66.0 (6.5)	58.9 (8.0)	H = 45.4	<0.001*
Initial surgery to the present (in months)		15.8 (3.9)	41.3 (11.9)	86.2 (18.1)	179.8 (54.4)	H = 109.0	<0.001*
BMI		24.0 (3.1)	24.3 (2.5)	24.3 (2.5)	21.8 (3.2)	H = 16.7	0.001*
Gender (%)	Female	17 (81.0)	26 (89.7)	24 (75.0)	33 (91.7)	χ² = 4.46	0.22
	Male	4 (19.0)	3 (10.3)	8 (25.0)	3 (8.3)		
Education (%)	Junior high school	5 (23.8)	8 (28.6)	9 (28.1)	5 (14.3)	χ² = 10.0	0.13
	High school	14 (66.7)	12 (42.9)	13 (40.6)	14 (40.0)		
	Higher education	2 (9.5)	8 (28.6)	10 (31.2)	16 (45.7)		
Bilateral surgery cases (%)		5 (23.8)	9 (31.0)	12 (37.5)	22 (61.1)	χ² = 9.96	0.02*

This study was approved (approval no. A-69) by the Ethics Committee of Sanjinkai Medical Corporation (Kasugai, Aichi, JPN). Informed consent was obtained from all participants. Participants were given a written or verbal explanation of the study, post which they provided informed consent to participate.

Data collection

Information regarding age, sex, BMI, the months from initial surgery to the present, educational history (junior high school, high school, and higher education), type of surgery (TKA or THA), and bilateral surgery status was obtained. Physical function was assessed by 10 physical therapists who were randomly assigned to evaluate physical function. The evaluations were conducted per the 2019 Asian Working Group for Sarcopenia (AWGS, 2019) guidelines [20].

Handgrip strength was determined by using a digital hand dynamometer (T.K.K. 401, Grip-D; Takei Scientific Instruments Co., Takei, Niigata, JPN). The 5-CS [19] was used alongside the SPPB [21], which measures muscle power (force × velocity) rather than pure strength. The SPPB has been reported to have high reliability, validity, and feasibility in the elderly population, with a maximum score of 12 indicating improved physical function [18]. This composite score allows for comparison with international studies using the same validated measure.

The one-leg standing test (OLST) was performed with the subject's eyes open and hands on the hips once per leg to assess balance control [22]. The test was recorded with a stopwatch to measure the time it took for the subjects to raise and lower their legs (maximum 60 seconds). The maximum time between the two measurements was recorded. The usual walking speed was determined by measuring the time it took for the subject to walk at a self-selected 'normal' pace along a 4 m walking path with a preliminary section, approximately 2 m, at the beginning and end.

The locomotive syndrome (LS) risk test was performed to identify a decline in mobility, which is closely related to disability [23]. The test consists of two functional assessments (the stand-up test [24] and the two-step test [25]) and a self-administered questionnaire (the GLFS-25; see Appendix A) [17]. Standardized reference values for these evaluation indices were established through epidemiological studies conducted in the Japanese population [11,22].

The stand-up test assessed lower extremity muscle strength [24]. Participants were required to stand up from stools of three different heights (20, 30, and 40 cm) using either a single-leg stand (SLS) or a both-leg stand (BLS). Success was determined by the ability to stand up and maintain posture for 3 seconds while standing. The tests were performed in the following order of increasing difficulty: BLS at 40 cm (easiest), BLS at 30 cm, BLS at 20 cm, and SLS at 40 cm (most difficult). The final score is based on the most challenging task that was successfully completed. The two-step test evaluates the maximum stride length in two steps [25]. The distance travelled between two steps was measured and divided by the height of the individual to obtain the two-step value.

The GLFS-25 is a self-administered questionnaire comprising 25 items [17]. These include four questions regarding physical pain experienced in the past month (Q1-4) and 21 questions about usual daily activities in the past month (Q5-25). Each item is rated on a 5-point scale from 0 (no impairment) to 4 (severe impairment). The total score ranges from 0 (no symptoms) to 100 (most severe symptoms).

The LS severity was evaluated using the LS risk test, which classifies individuals into three stages [22]. Stage 1 represents an early decline in mobility identified by meeting at least one of the following criteria: inability to stand on a single leg from a 40 cm stool, a two-step test score of <1.3, or a GLFS-25 score of ≥7. Stage 2 indicates a progressive decline identified by the inability to stand with both legs from a 20 cm stool, a two-step test score of <1.1, or a GLFS-25 score of ≥16. Stage 3 represents severe mobility limitations identified by an inability to stand with both legs from a 30 cm stool, a two-step test score of <0.9, or a GLFS-25 score of ≥24.

Statistical analysis

Participants were categorized into four groups based on the postoperative duration: short-term (<24 months, n=21), mid-term (24 to 59 months, n=29), long-term (60 to 119 months, n=32), and very long-term (≥120 months, n=36). Continuous data are presented as means and standard deviations (SD), and categorical data are presented as frequencies and percentages.

Between-group comparisons for continuous variables were conducted using the Kruskal-Wallis test, followed by the Steel-Dwass test for post-hoc analysis, which indicated a non-normal distribution of all variables. The effect sizes were calculated using epsilon squared (ε²) [15], with values of 0.01, 0.04, and 0.16, representing small, medium, and large effects, respectively. Chi-square tests or Fisher's exact tests were used for the analysis of categorical variables, and effect sizes were calculated using Cramer's V [16]. For binary categorical variables, such as surgery type and bilateral surgery status, Cramer's V values of 0.10, 0.30, and 0.50 were interpreted as indicating small, medium, and large effects, respectively. The Jonckheere-Terpstra test was used to examine trends in ordinal variables across the LS stage groups. For ordinal variables with three degrees of freedom (e.g., LS stage), Cramer's V values of 0.07, 0.21, and 0.35 were considered indicative of small, medium, and large effects, respectively.

The sample size calculation was based on the expected difference in the 5-CS test times between the short-term and very long-term groups. Assuming a moderate effect size (Cohen's d = 0.50), with an alpha level of 0.05 and a power of 0.80, a minimum of 20 participants per group was required. The final sample size of 21 to 36 participants per group provided adequate statistical power for primary analyses. For categorical analyses of LS stages, this sample size allowed for the detection of moderate associations (Cramer's V ≥ 0.24) with 80% power. All statistical analyses were performed using EZR version 1.34 (Saitama Medical Center, Jichi Medical University, Tochigi, JPN) [26], with statistical significance set at p<0.05.

Subgroup analyses were performed for THA patients (n=94) to address heterogeneity in surgery types. Multivariable linear regression models were constructed to examine the association between postoperative duration and functional outcomes. Model 1 was unadjusted; model 2 adjusted for age at surgery; model 3 additionally adjusted for BMI; and model 4 further adjusted for bilateral surgery status. Stratified analyses by bilateral surgery status were conducted to examine whether functional decline patterns differed between unilateral and bilateral cases. Adjusted regression coefficients with 95% confidence intervals were calculated. All models were tested for multicollinearity using variance inflation factors (VIF); VIF < 5 was considered acceptable.

## Results

A total of 118 patients were enrolled in this study and categorized into four groups based on the postoperative duration: short-term (<24 months, n=21), mid-term (24 to 59 months, n=29), long-term (60 to 119 months, n=32), and very long-term (≥120 months, n=36). Analysis of these groups revealed distinct patterns of changes in physical function and LS status across different postoperative periods.

Participant characteristics

The demographic and clinical characteristics showed significant variations across the groups (Table 1). While the mean age at evaluation was similar across all groups (approximately 73 to 74 years), the age at initial surgery differed significantly among groups (p<0.001), with the very long-term group being the youngest at surgery (58.9±8.0 years). The proportion of bilateral surgery cases showed a significant increasing trend with longer postoperative duration (p=0.02), reaching 61.1% in the very long-term group. Body mass index was significantly lower in the very long-term group (21.8±3.2) than in the other groups (p=0.001, ε²=0.140).

Physical function

Physical function analysis revealed specific patterns of change in the postoperative period (Table 2). The most notable change was observed in the 5-CS test, where the very long-term group required significantly more time (10.8 ± 3.5 s) than the short-term group (8.5 ± 2.4 s) (p=0.04, ε²=0.16). This represents a progressive decline in lower-extremity muscle function with longer postoperative duration. In contrast, the other physical function parameters remained relatively stable across the groups. No significant differences were observed in handgrip strength (p=0.13), one-leg standing time (p=0.33), usual walking speed (p=0.98), or the SPPB score (p=0.28).

**Table 2:** Physical function measurements by postoperative duration group Values are presented as mean (SD) unless otherwise indicated. The p-values were calculated using the Kruskal-Wallis test (H statistic) for continuous variables. *Significant difference between groups (p<0.05) 5-CS: Five-chair stand test; SPPB: Short Physical Performance Battery

Factor	Short term (<24 months)	Mid-term (24-59 months)	Long-term (60-119 months)	Very long-term (120 months)	Test statistic (H)	p-value
Number	21	29	32	36		
Hang grip strength (kg)	26.2 (8.3)	22.8 (5.8)	25.2 (8.0)	22.7 (4.7)	3.45	0.13
5-CS (s)	8.5 (2.4)	9.4 (1.9)	9.4 (2.5)	10.8 (3.5)	8.00	0.02*
One-leg standing time (s)	36.5 (21.9)	33.2 (19.7)	39.9 (20.5)	31.0 (20.8)	3.58	0.33
Usual walking speed (m/s)	1.1 (0.2)	1.1 (0.2)	1.1 (0.2)	1.1 (0.2)	0.49	0.98
SPPB	11.8 (0.7)	11.5 (0.9)	11.2 (1.4)	11.3 (1.0)	0.39	0.28

LS status

**Figure 1:**
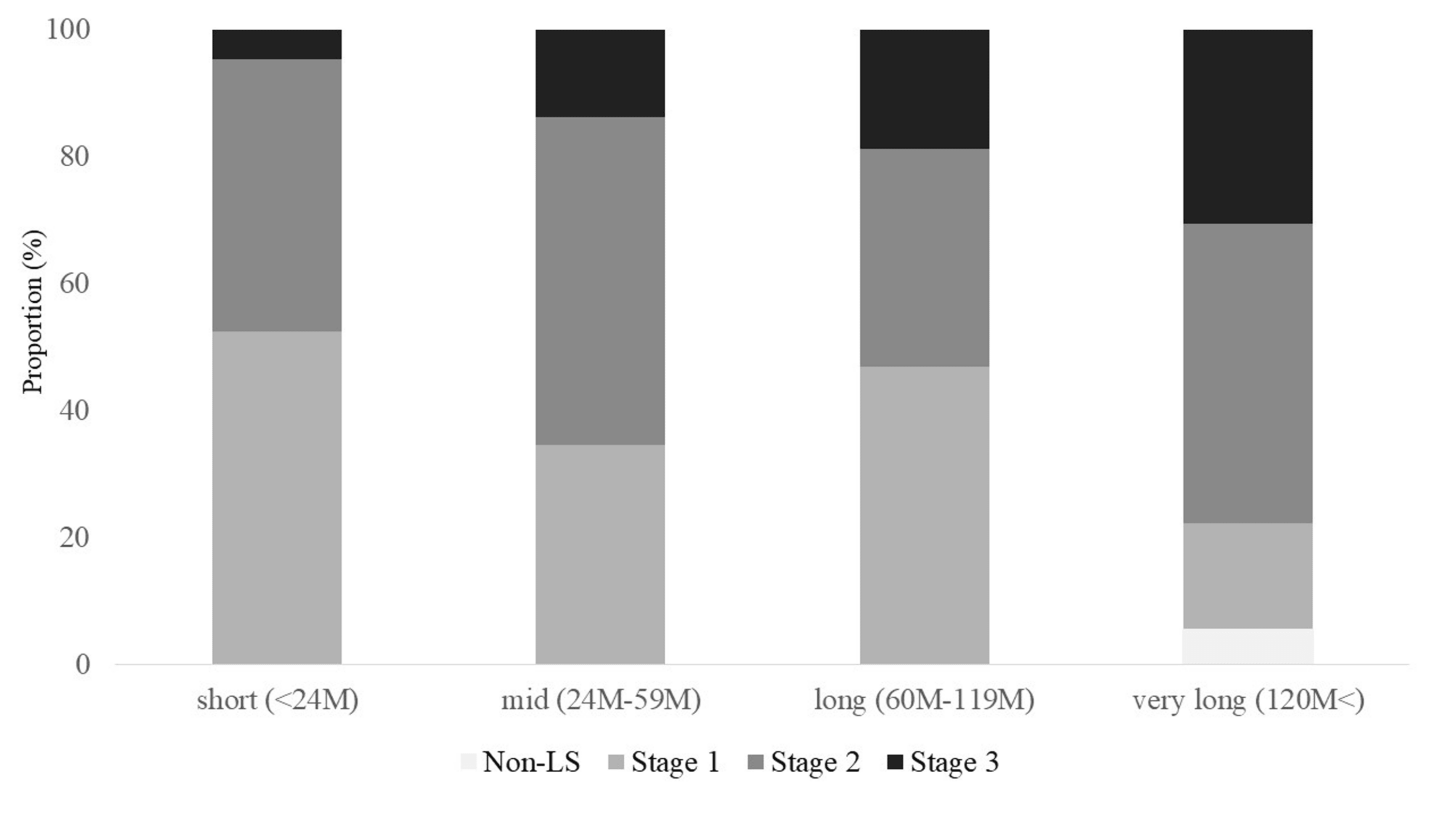
LS states and postoperative durations LS: Locomotive syndrome; M: Months

The severity and distribution of LS showed significant changes with increasing postoperative durations (Table 3). The proportion of severe cases (stage 3) progressively increased from 4.8% in the short-term group to 30.6% in the very long-term group (p=0.04, V=0.24, Figure 1). This trend was reflected in the GLFS-25 scores, which were significantly higher in the very long-term group (15.0 ± 9.0) than in the short-term group (8.5 ± 5.7) (p=0.03, ε²=0.15). Similarly, GLFS-25 scores in the long-term group (13.2 ± 12.1) exhibited a decreasing trend relative to the short-term group (9.0 ± 8.6) (p=0.03, ε²=0.13). However, no significant difference was observed between the long-term and very long-term groups (p=0.38, ε²=0.14). Analysis of individual LS components revealed varying patterns. While the stand-up test results showed no significant differences between groups (p=0.93), the two-step test scores showed a non-significant declining trend (p=0.07), from 1.2±0.1 in the short-term group to 1.1±0.2 in the very long-term group.

**Table 3:** LS status by postoperative duration group Values are presented as mean (SD) unless otherwise indicated. The p-values were calculated using the Kruskal-Wallis test (H statistic) for continuous variables and the chi-square test (χ² statistic) for categorical variables.*Significant difference between groups (p<0.05) LS: Locomotive syndrome; GLFS-25: 25-question Geriatric Locomotive Function Scale

Factor	Group	Short term (<24 months)	Mid-term (24-59 months)	Long-term (60-119 months)	Very long-term (120 months)	Test statistic (χ² or H)	p-value
Number		21	29	32	36		
LS stage	Non-LS	0 (0.0)	0 (0.0)	0 (0.0)	2 (5.6)	χ² = 14.0	0.04*
	Stage 1	11 (52.4)	10 (34.5)	15 (46.9)	6 (16.7)		
	Stage 2	9 (42.9)	15 (51.7)	11 (34.4)	17 (47.2)		
	Stage 3	1 (4.8)	4 (13.8)	6 (18.8)	11 (30.6)		
Stand-up test							
Single leg standing 40 cm (%)	Able	7 (33.3)	5 (17.2)	7 (21.9)	5 (13.9)	χ² = 3.33	0.34
	Unable	14 (66.7)	24 (82.8)	25 (78.1)	31 (86.1)		
Both legs standing 20 cm (%)	Able	14 (66.7)	18 (62.1)	20 (64.5)	18 (50.0)	χ² = 2.20	0.53
	Unable	7 (33.3)	11 (37.9)	11 (35.5)	18 (50.0)		
Both legs standing 30 cm (%)	Able	20 (95.2)	27 (93.1)	29 (90.6)	32 (88.9)	χ² = 0.82	0.84
	Unable	1 (4.8)	2 (6.9)	3 (9.4)	4 (11.1)		
Two-step test score (length of two strides/height)		1.2 (0.1)	1.2 (0.2)	1.1 (0.2)	1.1 (0.2)	H = 7.45	0.07
GLFS-25 score		8.5 (5.7)	13.2 (12.1)	9.0 (8.6)	15.0 (9.0)	H = 13.0	0.02*

Subgroup analysis in THA

Given the heterogeneity in surgery types across groups (p=0.03), we performed a focused analysis on THA patients (n=94). The demographic characteristics showed similar patterns to the overall cohort, with the very long-term group having the youngest age at surgery (58.4±7.8 years, p<0.001) and the highest prevalence of bilateral surgery (58.8%, p=0.003) (Table 4). In multivariable-adjusted analyses (Table 5), the very long-term group maintained significantly longer 5-CS times compared to the short-term group (β=1.77, 95% CI: 0.19-3.35, p=0.028) after adjusting for age at surgery, BMI, and bilateral surgery status. Similarly, GLFS-25 scores remained significantly higher in the very long-term group (β=9.97, 95% CI: 4.86-15.08, p<0.001). Bilateral surgery showed a trend toward worse 5-CS performance (β=1.10, p=0.09) but no significant association with GLFS-25 scores (β=1.99, p=0.33). Stratified analysis revealed different patterns between unilateral and bilateral THA patients. Among unilateral cases (n=59), the very long-term group showed a trend toward worse 5-CS performance (p=0.077) and significantly higher GLFS-25 scores (p=0.005) compared to the short-term group. In contrast, bilateral cases (n=35) showed no significant differences across postoperative periods, though interpretation is limited by small sample sizes in some groups.

**Table 4:** Subgroup analysis in THA patients featuring baseline characteristics and unadjusted comparisons Values are represented as mean (SD) or n (%). *Significant difference between groups (p<0.05) 5-CS: Five-chair stand test; SPPB: Short Physical Performance Battery; LS: Locomotive syndrome; GLFS-25: 25-question Geriatric Locomotive Function Scale

Factor	Group	Short term (<24 months)	Mid-term (24-59 months)	Long-term (60-119 months)	Very long-term (120 months)	p-value
Number		16	23	21	34	
Age (in years)		73.4 (4.7)	74.0 (6.0)	71.2 (5.4)	73.4 (6.2)	0.41
Age at initial surgery (in years)		72.1 (4.6)	70.7 (6.1)	63.7 (5.8)	58.5 (7.9)	<0.001*
Initial surgery to the present (in months)		14.9 (3.7)	38.7 (11.0)	88.86 (19.8)	182.2 (54.8)	<0.001*
BMI		23.6 (3.4)	23.9 (2.4)	23.47 (1.8)	21.7 (3.2)	0.01*
Gender (%)	Female	13 (81.2)	20 (87.0)	16 (76.2)	31 (91.2)	0.47
	Male	3 (18.8)	3 (13.0)	5 (23.8)	3 (8.8)	
Education (%)	1	2 (12.5)	5 (22.7)	3 (14.3)	4 (12.1)	0.17
	2	12 (75.0)	12 (54.5)	10 (47.6)	13 (39.4)	
	3	2 (12.5)	5 (22.7)	8 (38.1)	16 (48.5)	
Bilateral surgery cases (%)		1 (6.2)	7 (30.4)	7 (33.3)	20 (58.8)	0.003*
Hang grip strength (kg)		25.6 (8.7)	23.4 (6.0)	26.0 (7.6)	23.0 (4.7)	0.27
5-CS (s)		8.2 (2.0)	9.2 (1.9)	9.1 (2.1)	10.8 (3.5)	0.01*
One leg standing time (s)		43.0 (20.7)	35.4 (19.7)	45.9 (18.5)	31.3 (20.5)	0.04*
Usual walking speed (m/s)		1.1 (0.2)	1.1 (0.2)	1.1 (0.2)	1.1 (0.2)	0.79
SPPB		11.9 (0.3)	11.5 (1.0)	11.3 (1.6)	11.4 (0.9)	0.24
LS stage	Non-LS	0 (0.0)	0 (0.0)	0 (0.0)	1 (2.9)	0.03*
	Stage 1	9 (56.2)	9 (39.1)	14 (66.7)	6 (17.6)	
	Stage 2	6 (37.5)	11 (47.8)	6 (28.6)	17 (50.0)	
	Stage 3	1 (6.2)	3 (13.0)	1 (4.8)	10 (29.4)	
Stand-up test						
Single leg standing 40 cm (%)	Able	6 (37.5)	5 (21.7)	5 (23.8)	4 (11.8)	0.22
	Unable	10 (62.5)	18 (78.3)	16 (76.2)	30 (88.2)	
Both legs standing at 20 cm (%)	Able	11 (68.8)	17 (73.9)	17 (85.0)	18 (52.9)	0.09
	Unable	5 (31.2)	6 (26.1)	3 (15.0)	16 (47.1)	
Both legs standing at 30 cm (%)	Able	15 (93.8)	22 (95.7)	21 (100.0)	31 (91.2)	0.56
	Unable	1 (6.2)	1 (4.3)	0 (0.0)	3 (8.8)	

**Table 5:** Multivariable adjusted analysis and stratified results in THA patients *A p<0.05 indicates a significant difference. CI: Confidence interval; 5-CS: Five-chair stand test; GLFS-25: 25-question Geriatric Locomotive Function Scale

Analysis		Estimate (95% CI)	p-value
5-CS test (adjusted for age at surgery, BMI, bilateral surgery)	Short-term	Reference	-
	Mid-term	-0.10 (-1.80, 1.59)	0.90
	Long-term	-0.87 (-2.73, 0.98)	0.36
	Very long-term	1.77 (0.19, 3.35)	0.028*
	Bilateral surgery effect	1.09 (-0.16, 2.35)	0.09
GLFS-25 score (adjusted for age at surgery, BMI, bilateral surgery)	Short-term	Reference	-
	Mid-term	4.52 (-1.01, 10.04)	0.11
	Long-term	-1.43 (-7.55, 4.70)	0.64
	Very long-term	9.97 (4.86, 15.08)	<0.001*
	Bilateral surgery effect	1.99 (-2.05, 6.03)	0.33
Stratified analysis by bilateral surgery status	Unilateral THA (n=59)		
	5-CS: Very long-term vs short-term	1.89 (-0.19, 3.98)	0.08
	GLFS-25: Very long-term vs short-term	9.57 (2.99, 16.15)	0.005*
Bilateral THA (n=35)	5-CS: Very long-term vs short-term	1.05 (-1.06, 3.16)	0.33
	GLFS-25: Very long-term vs short-term	3.41 (-4.26, 11.07)	0.45

## Discussion

This cross-sectional study revealed three key findings regarding functional changes after total joint arthroplasty: a progressive decline in lower extremity muscle function, an increase in LS severity over time, and a high proportion of bilateral cases, particularly in the very long-term group. This decline remained significant after adjusting for potential confounders, including age at surgery, BMI, and bilateral surgery status (adjusted p=0.028), indicating that the observed deterioration is not solely attributable to patient characteristics but represents a time-dependent phenomenon.

The most notable finding was the marked decline in lower extremity muscle function, as evidenced by increased five-chair stand test times in the very long-term group (10.8±3.5 vs 8.5±2.4 seconds in short-term, p=0.04). This decline remained significant in THA patients after adjusting for age at surgery, BMI, and bilateral surgery status (adjusted p=0.028), and was observed even in unilateral THA cases (p=0.077), indicating that the observed deterioration represents a time-dependent phenomenon rather than being solely attributable to patient characteristics or bilateral surgery. The large effect size (ε²=0.16) indicated a clinically significant deterioration in muscle strength and power despite successful joint replacement. This finding is consistent with previous studies reporting persistent functional deficits in long-term arthroplasty patients compared with healthy controls [27] but also suggests that this decline may be more substantial than previously recognized. Notably, basic mobility functions such as walking speed and balance (one-leg standing time) remained relatively stable across all postoperative periods. This differential pattern suggests that, while basic mobility is maintained after arthroplasty, more demanding tasks requiring muscle power show significant susceptibility to decline over the long-term postoperative period. The association between 5-CS performance and LS severity, previously documented in community-dwelling older adults [28], further emphasizes the clinical importance of this finding and its implications for long-term rehabilitation strategies. Our subgroup analysis of THA patients provides additional insights into the mechanisms of functional decline. The persistence of significant differences after comprehensive adjustment suggests that time-dependent deterioration is not merely an artifact of confounding factors. The stronger associations observed in unilateral cases paradoxically support this interpretation, as these patients theoretically have better compensatory mechanisms through their unaffected limb.

The second key finding was the significant increase in LS severity over a longer postoperative period, as demonstrated by a moderate effect size (V=0.24). The proportion of severe cases (stage 3) increased from 4.8% in the short-term group to 30.6% in the very long-term group, with GLFS-25 scores showing substantial changes (ε²=0.15). The moderate-to-large effect sizes for both LS stage progression and GLFS-25 scores suggest that these changes represent meaningful clinical deterioration. The prevalence of LS in our cohort (98.3%; mean age, 73 years; mean follow-up, 91.2 months) exceeded previous reports of 88.9% in TKA patients [12] and 83.0% in THA patients [13] at two years post-surgery. This higher prevalence suggests the need for extended monitoring beyond the immediate postoperative period [8] and indicates that current rehabilitation protocols may require modifications to address long-term functional decline. The prevalence of LS in our cohort (98.3%) was notably higher than that reported in previous studies that focused solely on GLFS-25 assessment in long-term THA patients (27.7%) [29]. This higher prevalence likely reflects our comprehensive LS assessment using all three components of the official diagnostic criteria: the stand-up test, two-step test, and GLFS-25. Moreover, the GLFS-25 questionnaire's multidimensional nature, encompassing pain (four items), activities of daily living (16 items), social function (three items), and mental health (two items), suggests that the observed functional decline extends beyond physical limitations to affect broader aspects of patients' daily lives and social participation. This comprehensive impact of functional decline aligns with previous research identifying older age, extended follow-up periods, and greater comorbidities as negative prognostic factors for sustained functional outcomes after arthroplasty [30].

The high proportion of bilateral surgery cases in the very long-term group (61.1%, V=0.290) warrants careful interpretation. This characteristic likely reflects the natural history of developmental hip dysplasia common in Japan [31,32]. While bilateral surgery was associated with a trend toward worse 5-CS performance (β=1.10, p=0.09), our stratified analysis demonstrated that functional decline occurs even in unilateral cases. Specifically, unilateral THA patients showed similar patterns of deterioration in both 5-CS performance and GLFS-25 scores, supporting that time-related functional decline is not merely an artifact of bilateral surgery prevalence.

This study has several limitations. First, the cross-sectional design prevents establishing causal relationships. Second, the disproportionate distribution of THA (n=94) versus TKA (n=24) cases limits procedure-specific conclusions. Third, the higher prevalence of bilateral surgery in the very long-term group may introduce selection bias. However, our comprehensive adjustment for confounders and consistent findings in unilateral subgroup analyses support the robustness of our conclusions. Fourth, the absence of non-arthroplasty control groups limits our ability to distinguish surgery-related changes from normal aging processes.

Future studies should address these limitations through longitudinal studies that track functional changes and LS progression separately in patients undergoing THA and TKA. Investigation of factors contributing to maintained function in patients without LS in the very long-term group would be valuable for developing preventive strategies. Additionally, studies examining the relationship between bilateral surgery timing and functional outcomes are needed to optimize the long-term management strategies.

Based on our findings, we propose specific preventive strategies for long-term management after joint arthroplasty. First, regular monitoring of muscle power using the 5-CS test is recommended, particularly beyond five years post-surgery, as this simple test effectively identifies early functional decline. Second, a comprehensive assessment using all three components of the LS evaluation should be conducted annually, as our results indicate that relying solely on the GLFS-25 may underestimate functional deterioration. Third, targeted intervention programs should focus on progressive resistance training of the lower limb muscles, as the deterioration in muscle power-dependent functions appears more pronounced than basic mobility. In bilateral cases, which showed a higher prevalence in our study, special attention should be paid to symmetrical functional recovery and balanced muscle strength. These interventions should be initiated before the five-year postoperative period, when our data suggest that functional decline begins to accelerate.

## Conclusions

This study demonstrates that while joint arthroplasty maintains basic mobility functions such as walking speed over extended periods, there is a significant time-dependent deterioration in muscle power-dependent functions and LS severity, particularly beyond 10 years post-surgery. Our adjusted analyses and subgroup findings in unilateral cases confirm that this decline is not merely attributable to patient characteristics or bilateral surgery status. We recommend implementing routine functional assessments using the 5-CS test and comprehensive LS evaluation, particularly after five years post-surgery when functional decline begins to accelerate. Targeted resistance training programs focusing on lower limb muscle power should be considered as part of long-term postoperative management to preserve functional capacity and quality of life in the growing population of long-term arthroplasty survivors.

## Appendices

Appendix A

Table 6 features the English translation of the GLFS-25. 

**Table 6:** The GLFS-25 questionnaire items The English version of the GLFS-25 questionnaire items listed above is sourced from the Japanese Orthopaedic Association's official LS awareness website (https://locomo-joa.jp/check/test/locomo25/), Locomo Online, which is publicly available for health checks. GLFS-25: 25-question Geriatric Locomotive Function Scale, LS: Locomotive syndrome

Instructions: Please answer the following 25 questions about your physical condition and daily life over the past month. Each item is rated on a 5-point scale from 0 (no impairment) to 4 (severe impairment).
Questionnaire item
Questions regarding pain in the last month:
1. Have you had any pain (including numbness) in your neck or upper limbs?
2. Have you had any pain in your back, lower back or buttocks?
3. Have you had any pain (including numbness) in your lower limbs?
4. To what extent has it been painful to move your body in daily life?
Questions regarding activities of daily living in the last month:
5. To what extent has it been difficult to get up from a bed or lie down?
6. To what extent has it been difficult to stand up from a chair?
7. To what extent has it been difficult to walk inside the house?
8. To what extent has it been difficult to put on and take off a shirt?
9. To what extent has it been difficult to put on and take off trousers and pants?
10. To what extent has it been difficult to use the toilet?
11. To what extent has it been difficult to wash your body in the bath?
12. To what extent has it been difficult to go up and down the stairs?
13. To what extent has it been difficult to walk briskly?
14. To what extent has it been difficult to keep yourself neat?
15. How far can you keep walking without rest?
16. To what extent has it been difficult to go out to visit neighbors?
17. To what extent has it been difficult to carry objects weighing 2 kg?
18. To what extent has it been difficult to go out using public transportation?
19. To what extent have simple tasks and housework been difficult?
20. To what extent have load-bearing tasks and housework been difficult?
21. To what extent has it been difficult to perform sports activities?
22. Have you felt restricted from meeting your friends?
23. Have you felt restricted from joining social activities?
24. Have you ever felt anxious about falls in your house?
25. Have you ever felt anxious about being unable to walk in the future?
